# Functional Connectome Signatures of Patients with Asymptomatic and Typical Alzheimer’s

**DOI:** 10.1002/alz70862_110031

**Published:** 2025-12-23

**Authors:** Xiaoqing Huang, Rishit Puri, Dayu Sun, Yi Zhao, Jie Zhang, Kun Huang, Yijie Wang

**Affiliations:** ^1^ Indiana University, School of Medicine, Indianapolis, IN USA; ^2^ Indiana University, Department of Computer Science, Bloomington, IN USA; ^3^ Indiana University School of Medicine and The Richard M. Fairbanks School of Public Health, Indianapolis, IN USA; ^4^ INDIANA UNIVERSITY, Indianapolis, IN USA; ^5^ Center for Comp. Biology and Bioinformatics, Indiana University, School of Medicine, Indianapolis, IN USA; ^6^ Indiana University, Department of Computer Science, Carmel, IN USA

## Abstract

**Background:**

Functional magnetic resonance imaging (fMRI) has emerged as a powerful modality for investigating brain activity, offering superior spatial resolution and a non‐invasive means to probe functional connectivity. In the context of Alzheimer’s disease (AD), studying these disruptions is crucial for understanding how functional connectivity changes in salient brain activation networks for different subtypes. This study compares asymptomatic and typical AD groups to identify early alterations in brain networks that may inform future diagnostic and therapeutic strategies.

**Method:**

Three analytical pipelines were employed to characterize functional connectivity comprehensively from ADNI resting state fMRI data. The first pipeline parcellated the brain using functional atlases, then edge quantification through Graphical Lasso and group sparse covariance estimation, thereby capturing inter‐regional dependencies, using Nilearn. The second pipeline used independent component analysis (ICA) to decompose the fMRI data into distinct spatial components; mutual information was then applied to quantify the statistical relationships among these components. The third pipeline utilized FSL to perform advanced brain decomposition techniques like ICA and dual regression to generate time series that were subsequently analyzed to discern significant connectivity patterns between asymptomatic and typical AD cohorts.

**Result:**

Across all pipelines, heatmaps were generated to visualize regions of high and low brain activity, while network diagrams demonstrated varying levels of connectivity strength and hub distribution. For instance, when compared to typical AD, patients having asymptomatic AD have more regions with negative covariance. These regions are the right posterior temporal region, the default mode network. On the contrary, the regions with high positive connections or brain activity for typical AD are the auditory cortex, intraparietal sulcus, dorsal, ventral anterior cingulate cortex, and left lateral occipital complex. The posterior occipital region has strong negative connections with other regions of the brain in Typical AD.

**Conclusion:**

This study underscores the utility of fMRI‐based techniques for elucidating connectivity differences in asymptomatic and symptomatic stages of AD. By mapping out network‐level alterations, the resulting brain graphs offer valuable insights into the pathophysiology of AD, highlighting regions and pathways that may be critical in the early detection and treatment of the disease, potentially in a clinical setting utilizing fMRI as a biomarker.

